# Occupation-modulated language networks and its lateralization: A resting-state fMRI study of seafarers

**DOI:** 10.3389/fnhum.2023.1095413

**Published:** 2023-03-13

**Authors:** Huijun Wu, Deyuan Peng, Hongjie Yan, Yang Yang, Min Xu, Weiming Zeng, Chunqi Chang, Nizhuan Wang

**Affiliations:** ^1^School of Biomedical Engineering, Health Science Center, Shenzhen University, Shenzhen, China; ^2^Department of Neurology, Affiliated Lianyungang Hospital of Xuzhou Medical University, Lianyungang, China; ^3^CAS Key Laboratory of Behavioral Science, Center for Brain Science and Learning Difficulties, Institute of Psychology, Chinese Academy of Sciences, Beijing, China; ^4^Center for Brain Disorders and Cognitive Science, Shenzhen University, Shenzhen, China; ^5^Lab of Digital Image and Intelligent Computation, Shanghai Maritime University, Shanghai, China; ^6^Peng Cheng Laboratory, Shenzhen, China; ^7^School of Biomedical Engineering, ShanghaiTech University, Shanghai, China

**Keywords:** functional magnetic resonance imaging, lateralization, occupational neuroplasticity, occupation, language network, seafarers

## Abstract

**Introduction:**

Studies have revealed that the language network of Broca’s area and Wernicke’s area is modulated by factors such as disease, gender, aging, and handedness. However, how occupational factors modulate the language network remains unclear.

**Methods:**

In this study, taking professional seafarers as an example, we explored the resting-state functional connectivity (RSFC) of the language network with seeds (the original and flipped Broca’s area and Wernicke’s area).

**Results:**

The results showed seafarers had weakened RSFC of Broca’s area with the left superior/middle frontal gyrus and left precentral gyrus, and enhanced RSFC of Wernicke’s area with the cingulate and precuneus. Further, seafarers had a less right-lateralized RSFC with Broca’s area in the left inferior frontal gyrus, while the controls showed a left-lateralized RSFC pattern in Broca’s area and a right-lateralized one in Wernicke’s area. Moreover, seafarers displayed stronger RSFC with the left seeds of Broca’s area and Wernicke’s area.

**Discussion:**

These findings suggest that years of working experience significantly modulates the RSFC of language networks and their lateralization, providing rich insights into language networks and occupational neuroplasticity.

## 1. Introduction

The human brain controls language (Catani et al., 2005), movement (Weiller et al., 1996), learning (Hein et al., 2016), emotion (Kringelbach and Berridge, 2017), memory (Lane et al., 2015), consciousness (Penfield, 2015), the subconscious (Martin et al., 2016), and other high-level cognitive activities. Language-related functions were among the first to be ascribed to a specific location in the human brain (Broca, 1861) and have been the subject of intense research for well over a century. A “classical model” of language organization, based on data from aphasic patients with brain lesions, was popularized during the late 19th century and remains in common use (Wernicke, 1874; Mayeux and Kandel, 1985). Furthermore, studies with neuroimaging techniques (Biswal et al., 1995; Achard et al., 2006; Damoiseaux et al., 2006) have found that the human brain has stable, low-frequency fluctuations in resting states, forming reliable intrinsic brain networks (Wang et al., 2012, 2013, 2015a,b, 2016, 2017a; Yao et al., 2013; Shi et al., 2017) with specific functions, such as the language network (Gohel et al., 2019; Broday-Dvir and Malach, 2021), auditory network (Chen et al., 2017), default network (Schilbach et al., 2016), and visual network (Shen et al., 2019).

Resting-state functional magnetic resonance imaging (rsfMRI) is widely used to map the physiology and behavior of the healthy/diseased brain (Lottman et al., 2019). Furthermore, rsfMRI-based resting-state functional connectivity (RSFC) provides a useful technique for assessing lateralization, which is increasingly being used in clinical practice and research (Fox and Greicius, 2010; Friederici, 2011). Previous studies on the neurophysiological basis of human language ability have generally found that, for most individuals, the left hemisphere is the dominant hemisphere of language ability (Bradshaw et al., 2017). For example, clinical language lateralization assessment is necessary in the examination of epilepsy patients prior to resection surgery of the temporal lobe (Baxendale, 2009). At the same time, in the healthy population, language lateralization has historically been found to depend on gender (Nenert et al., 2017), age (Sepeta et al., 2016), handedness (Agcaoglu et al., 2021), genetics (Schmitz et al., 2017), and language-learning experience (Gurunandan et al., 2020). However, language lateralization may also be affected by other factors, e.g., occupation, which is the main focus in this study. At present, only a few research studies have reported on the association of the language network and occupational neuroplasticity (Villar-Rodríguez et al., 2020; Wu et al., 2020). Villar-Rodríguez et al. (2020) found musicianship is related to atypical (symmetric or right-hemispheric) language dominance in healthy left-handed subjects. Further, the lateralization of language functions can be used to explain subtle differences in behavior and cognitive levels (Szaflarski et al., 2006; Olulade et al., 2020). Thus, given the mechanism of language lateralization and the relative noise (auditory stimulation, e.g., the sound of waves or machines) and isolation (lacking social interaction) of seafarers’ long-term training and stable work environments, we hypothesized that there is a higher incidence of atypical language dominance among seafarers.

In this paper, taking seafarers as an example, two important sub-functions of language, namely, the language network (study 1) and the lateralization of the language network (study 2), were investigated to explore the association between the occupational factor and language by using the method of resting state functional connectivity (RSFC) (Tomasi and Volkow, 2012; Zhu et al., 2014). The analysis is presented together with interpretations, discussion, and conclusions related to the language network and occupational neuroplasticity in professional seafarers.

## 2. Materials and methods

### 2.1. Data acquisition

Since seafarers have been engaged in repetitive technical work for a long time we recruited twenty male professional seafarers (age: 42–57 years, mean age = 49 years old, right handedness) from a shipping company in Shanghai, China. All of them had more than 10 years of experience in navigation. For non-seafarers, 20 Chinese male participants (age: 48–55 years, mean age = 51 years old, right handedness), were recruited from land-based jobs (i.e., campus landscaping and office support) at university or secondary school campuses. All the subjects in the non-seafarer group had no maritime professional training, maritime navigational skills, or long-term experience on the sea. All subjects signed the informed consent form and were considered to have normal functions of language and communication. Also, no history of mental health conditions or neurological diseases were reported. The blood-oxygen-level-dependent imaging (BOLD) rsfMRI data for each participant was scanned at the Shanghai Key Laboratory of Magnetic Resonance. All participants were informed about the purpose of the study and signed a written consent form according to the procedures approved by the IRB of East China Normal University (ECNU). The specific parameters were listed as follows: GE 3.0 Tesla using a gradient echo EPI, a total of 36 slices covering the whole brain area, 160 time points, TR (time of repetition) = 2 s, matrix size = 64 × 64, in-plane resolution = 3.75 mm × 3.75 mm, and slice thickness = 4 mm. The detailed information related to the dataset can also be found in Wang et al. (2017b), Wang et al. (2018), Shi et al. (2021), and Yan et al. (2022).

### 2.2. Data preprocessing

All data preprocessing was performed using the Data Processing Assistant for RS-fMRI software package (DPARSF) (Yan and Zang, 2010) which is based on Statistical Parametric Mapping (SPM)^1^ and the Resting-State fMRI Data Analysis Toolkit (REST).^2^ The preprocessing steps for the Resting-State fMRI data of each subject were as follows: (1) slice timing; (2) realignment; (3) normalization by EPI template (resampling voxel size = 3 mm*3 mm*3 mm); (4) spatial smoothing using a Gaussian kernel with FWHM = 6 mm; (5) nuisance regression including covariates such as six head motion parameters, whole brain mean signal, white matter signal, and cerebrospinal fluid signal; (6) band-pass temporal filtering (0.01–0.1 Hz); and (7) scrubbing volumes with sudden head motion, i.e., a threshold of frame-wise displacement (FD) was set to 0.05, and we removed one volume before and two volumes after the motion spike.

### 2.3. Functional connectivity and lateralization of language network

The RSFC method generates a high-precision functional connection diagram of a complex brain system by interpreting the relevant patterns of low-frequency fluctuations in the blood oxygen level signals, which can be used to identify language-related functional tissues. In this paper, the Broca and Wernicke in the left side of brain were selected as the region of interest (ROI) (lBro and lWer), with MNI coordinates (−51, 27, 18) and (−51, −51, 30) as the center of the seed points (Zuo et al., 2013; Zhu et al., 2014) with a radius of 3 mm, respectively. In order to explore the functional asymmetry of the main language regions in the brain, the right Broca’s area (rBro) and right Wernicke’s area (rWer) were reversed from the left side of the brain to the right side, respectively; the central coordinates of the seed points, i.e., rBro and rWer, were (51, 27, 18) and (51, −51, 30), with the same volume size. Based on DPARSF software, the time series of the four aforementioned ROIs were extracted, and then the Pearson correlation coefficients were computed between the average time series of four ROIs and the time series of each voxel across the brain. Furthermore, the correlation coefficient (cc) value was subjected to Fisher Z-transformation (Fisher, 1921) according to formula (1). According to the transformed correlation coefficient, the functional connection diagram of the seed points and whole brain voxels can be obtained according to the following formula:


(1) $$z=\frac{1}{2}\,l\,n\,\left(\frac{1+c\,c}{1-c\,c}\right)$$

**FIGURE 1:**
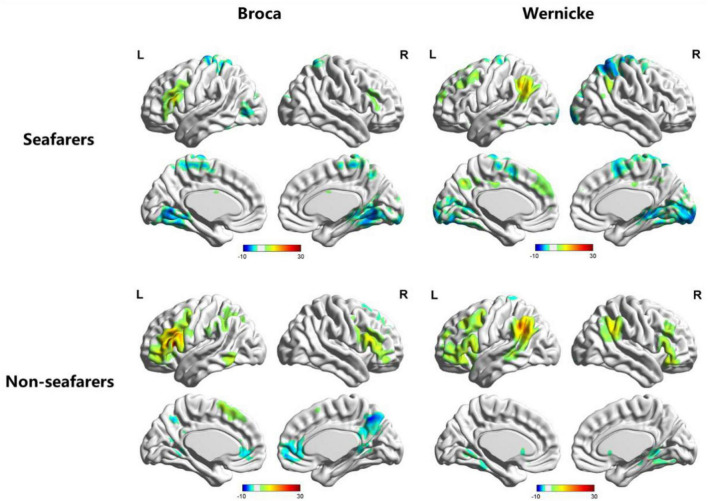
Visualization of functional connectivity using Broca’s region and Wernicke’s region as the independent seeds in the seafarer and non-seafarer group (*p* 0.005, cluster size >200 voxels, corresponding to corrected *p*_*FWE*_ 0.05). R: right hemisphere, L: left hemisphere.

**FIGURE 2:**
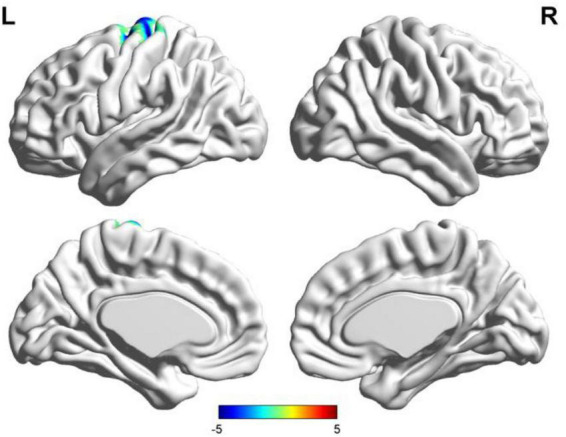
Results of two-sample *t*-test of functional connectivity of Broca’s region (the seafarer group < the non-seafarer group; *p* 0.005, cluster size >200 voxels, corresponding to corrected *p*_*FWE*_ 0.05). R: right hemisphere, L: left hemisphere.

**FIGURE 3:**
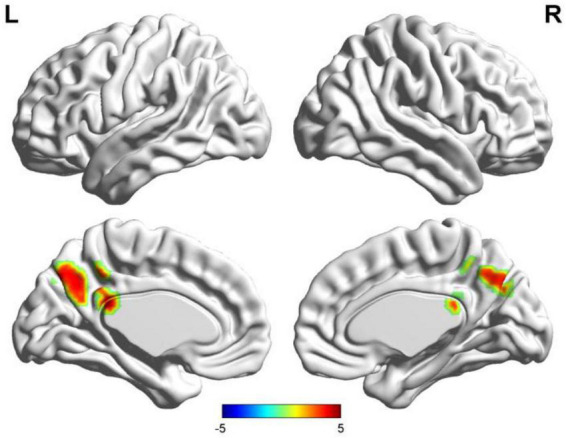
Results of two-sample *t*-test of functional connectivity of Wernicke’s region (the seafarer group > the non-seafarer group; *p* 0.005, cluster size >200 voxels, corresponding to corrected *p*_*FWE*_ 0.05). R: right hemisphere, L: left hemisphere.

Based on the RSFC map of each subject, the one-sample *t*-test results (Figure 1) for each group and two-sample *t*-test results (Table 1; Figures 2, 3) between two groups were performed using REST software.

**TABLE 1:** The cerebral cortex involved significant functional connectivity with Broca’s and Wernicke’s areas in the seafarer group and the non-seafarer group (*p* 0.005, cluster size >200 voxels, corresponding to corrected *p*_*FWE*_ 0.05).

No.	Anatomical region	BA	MNI coordinates			Peak value
			**X**	**Y**	**Z**	
**Broca’s area (seafarer < non-seafarer)**						
1	L SFG	6	−28	−8	68	-3.78
2	Precentral gyrus	6	−32	−22	67	-4.803
3	Precentral gyrus	4	−33	−28	68	-3.74
4	L MFG	6	−35	−7	58	-2.559
**Wernicke’s area (seafarer > non-seafarer)**						
1	Parietal	7	−2	−65	41	3.465
2	Limbic	31	−5	−43	41	3.701
3	Precuneus	7	11	−69	35	4.016
4	Cingulate gyrus	23	−5	−37	25	4.528

BA, Brodmann area; L, left; SFG, superior frontal gyrus; MFG, middle frontal gyrus.

When discussing the functional asymmetry, we calculated each seed-based whole brain RSFC maps, namely the lBro and lWer RSFC maps, and the flipped rBro and rWer RSFC maps. The hemispheric asymmetry was evaluated through comparison of the RSFC maps of the lBro and lWer and the left-right flipped RSFC maps of the rBro and rWer (Yan et al., 2009). In this study, the non-normalized asymmetry index (*AI*) is defined by following formula (2) (Zhu et al., 2014):


(2) $$A\,I=z\,F\,C_{L}-z\,F\,C_{f\,l\,i\,p\,p\,e\,d\,R},$$

where *zFC*_*L*_ is a whole brain functional connection diagram based on the left seed points (i.e., lBro and lWer), respectively, and *zFC*_*flipped R*_ is a left-right flipped functional connection diagram based on the right seed points (rBro and rWer), respectively. Similarly, as in previous studies (Yan et al., 2009; Zhu et al., 2014), ipsilateral asymmetry was shown on the left side of the *AI* map, representing the difference between the lBro or lWer and the left hemisphere (LH) and the rBro and rWer and the right hemisphere (RH). Also, contralateral asymmetry was established on the right side of the *AI* map, indicating the differences between the lBro or lWer and the RH and the rBro and rWer and the LH. Further, a one-sample *t*-test was conducted to reveal regions which show significant hemispheric asymmetry based on individual *AI* maps. Moreover, the two-sample *t*-test was applied to analyze the differences of language lateralization between seafarers and the control participants. All RSFC maps and *AI* maps were established with the test criteria of *p* 0.005 and cluster size >200 voxels (corresponding to corrected *p*_*FWE*_ 0.05).

## 3. Results

### 3.1. Functional connectivity using language areas as seed points

First, we identified the brain areas that were significantly functionally correlated with the two seed points, i.e., Broca’s and Wernicke’s regions, with regard to the seafarer group and the non-seafarer group; the results are shown in Figure 1, and the color bar reflects the correlation. Figure 1 shows that the linguistic functional connectivity patterns of the non-seafarer group were highly similar to those previously reported (Zhu et al., 2014), while the ones of seafarers showed some differences in the involved locations and connectivity values. Further, two-sample *t*-test analysis of the linguistic functional connectivity patterns from the seafarer and non-seafarer groups revealed that: the negative functional connectivity of Broca’s region appeared weaker in the seafarer group than the non-seafarer group, especially in the left superior/middle frontal gyrus and left precentral gyrus (Figure 2; Table 1); functional connectivity of the Wernicke’s region as the seed region was higher in the seafarer group compared to the non-seafarer group (Figure 3; Table 1), where this phenomenon was especially reflected in the posterior cingulate cortex and precuneus.

### 3.2. Functional asymmetry of language areas

**FIGURE 4:**
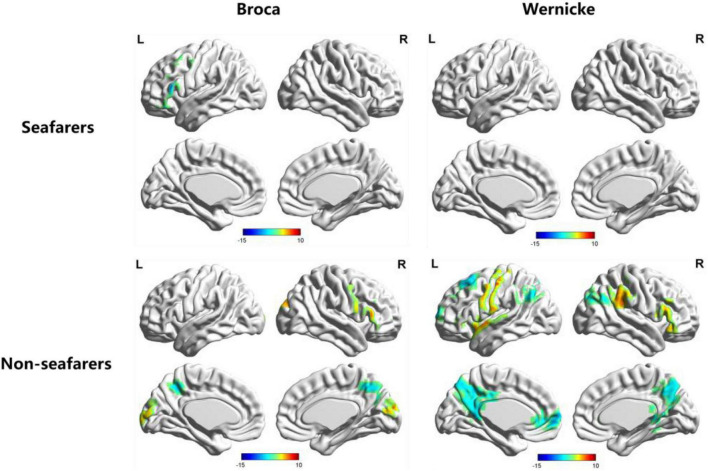
*AI* maps with regard to functional lateralization of language network in the seafarer and non-seafarer groups, respectively (*p* 0.005, cluster size >200 voxels, corresponding to corrected *p*_*FWE*_ 0.05). R: right hemisphere; L: left hemisphere.

**FIGURE 5:**
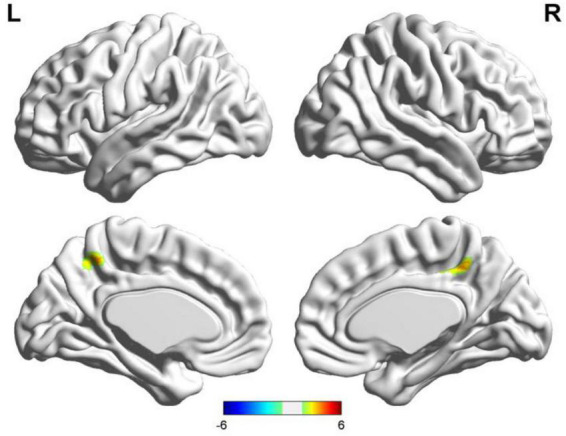
Results of two-sample *t*-test of language lateralization in terms of Broca’s area (the seafarer group > the non-seafarer group; *p* 0.005, cluster size >200 voxels, corresponding to corrected *p*_*FWE*_ 0.05). R: right hemisphere, L: left hemisphere.

**FIGURE 6:**
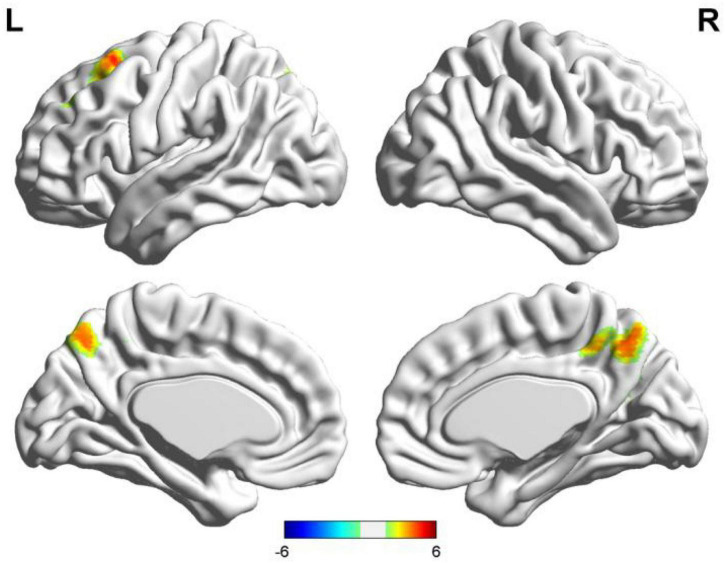
Results of two-sample *t*-test of language lateralization in terms of Wernicke’s area (the seafarer group > the non-seafarer group; *p* 0.005, cluster size >200 voxels, corresponding to corrected *p*_*FWE*_ 0.05). R: right hemisphere, L: left hemisphere.

A profile of the lateralization for each subject was obtained based on the RSFC map in terms of Broca’s area and Wernicke’s area. REST software was used to conduct a one-sample *t*-test (results in Table 2 and Figure 4) for each group and a two-sample *t*-test (results in Table 3 and Figures 5, 6) between the two groups, respectively, where the test criterion was *p* < 0.005 and cluster size >200 voxels (corresponding to corrected *p*_*FWE*_ < 0.05).

**TABLE 2:** The cerebral regions involving significant functional lateralization with Broca’s and Wernicke’s areas in the seafarer group and non-seafarer group (*p* 0.005, cluster size >200 voxels, corresponding to corrected *p*_*FWE*_ 0.05).

No.	Anatomical regions	BA	MNI coordinates			Peak value
			**X**	**Y**	**Z**	
**Non-seafarer**						
**Broca’s area**						
1	R IFG	45	57	27	27	10.839
2	L IFG	45	−57	27	21	-16.882
3	R SOG	18	15	−90	18	5.1994
4	B precuneus	7	3	−57	51	-5.0739
**Wernicke’s area**						
1	L STG	6	−57	−15	54	9.0572
2	L cuneus/precuneus	23	−21	−54	24	-10.3471
3	L SFG	10	−18	63	9	-5.9298
4	R IFG	47	45	30	−3	6.042
5	R SMG	40	63	−54	36	8.331
6	L MFG / SFG	8/9	−27	18	63	-14.023
**Seafarer**						
**Broca’s area**						
1	L IFG	45	−57	27	18	-14.4288
**Wernicke’s area**						
**There is no cluster!**						

BA, Brodmann area; L, left; R, right; B, bilateral; SFG, superior frontal gyrus; MFG, middle frontal gyrus; IFG, inferior frontal gyrus; STG, superior temporal gyrus; SMG, supramarginal gyrus; SOG, superior occipital gyrus.

**TABLE 3:** The cerebral regions involved significant functional lateralization with regard to Broca’s and Wernicke’s areas (the seafarer group > the non-seafarer group; *p* 0.005, cluster size >200 voxels, corresponding to corrected *p*_*FWE*_ 0.05).

No.	Anatomical regions	BA	MNI coordinates			Peak value
			**X**	**Y**	**Z**	
**Broca’s area**						
1	B precuneus	7	−13	−54	46	3.937
2	B paracentral lobule	31	6	−34	45	4.016
3	B middle cingulum	24	6	−18	40	3.189
**Wernicke’s area**						
1	B precuneus	7	−9	−72	42	3.858
2	L SFG	8	−23	22	56	4.764
3	L MFG	6	−23	20	59	5.551

BA, Brodmann area; L, left; B, bilateral; SFG, superior frontal gyrus; MFG, middle frontal gyrus.

According to Figure 4, three distinct cortical language-related areas were observed in the left hemisphere. These were: (1) for the Broca’s region of the non-seafarer group, significant ipsilateral asymmetry showed in the left inferior frontal gyrus (IFG) and precuneus, while contralateral asymmetry was displayed in the right superior occipital gyrus (SOG), IFG, and precuneus; (2) for the Wernicke’s region of the non-seafarer group, significant ipsilateral asymmetry areas were in the left superior frontal gyrus (SFG), middle frontal gyrus (MFG), precentral, precuneus, and cuneus, while the right IFG and supramarginal gyrus (SMG) showed contralateral hemispheric asymmetry; (3) for the Broca’s region of seafarer group, the IFG showed significant ipsilateral hemispheric asymmetry and greater connection with rBro; (4) there was no significant language networks’ lateralized brain areas for the Wernicke’s area of the seafarer group. The detailed brain regions involved in significant functional lateralization with regard to Broca’s and Wernicke’s areas for the seafarer and control groups were coordinated and recorded in Table 2.

In order to further quantify the differences in language lateralization between the seafarer group and the non-seafarer group, we performed a two-sample *t*-test analysis on the *AI* maps of the two core language regions for the two groups. The statistical results are shown in Figures 5, 6 and Table 3. For the *AI* maps corresponding to the Broca’s region, the seafarer group elicited greater functional asymmetry in the paracentral and precuneus (BA7 and BA31). For the *AI* maps corresponding to the Wernicke’s area, the lateralization difference between the seafarer group and the non-seafarer group was mainly reflected in the left frontal gyrus and bilateral precuneus.

## 4. Discussion

Previous studies have investigated whole-brain language networks using the RSFC method (Gao et al., 2019; Sulpizio et al., 2020). Sulpizio et al. (2020) found that each experience-related factor seems to play a role in brain plasticity changing; bilingual experience especially impacts both within and between language and control networks. Interestingly, the functional connectivity of language networks is also affected by disease, and children with autism spectrum disorders (ASDs) show increased connectivity between regions of an extended language network. Further, these brain regions are associated with self-reflection and visual processing (Gao et al., 2019). In its most general form, this model proposes a frontal “expressive” area for planning and executing speech and writing movements, named after Broca (1861), and a posterior “receptive” area for analysis and identification of linguistic sensory stimuli, named after Wernicke (1874). One study (Binder et al., 1997) suggested that Wernicke’s area, although important for auditory processing, is not the primary location where language comprehension occurs, and that the frontal areas involved in language extend well beyond the traditional Broca’s area to include much of the lateral and medial prefrontal cortex. Based on seed regions in Broca and Wernicke, seed-based RSFC were applied to the characterization and reproducibility of functional connectivity of language networks (Tomasi and Volkow, 2012; Zhu et al., 2014; Wang et al., 2019). Meanwhile, language lateralization has been widely approached to detect different patterns in children (Phillips et al., 2021; Stipdonk et al., 2021), tumors (Połczyńska et al., 2021), psychiatric disorders (Jouravlev et al., 2020), and neurological disorders (Rolinski et al., 2020).

### 4.1. Language network’s functional connectivity and its relation to occupation

In this study, we selected the special occupation group of seafarers as the research object, and compared the functional language network seafarers with non-seafarers. Regarding the seafarers, the functional connectivity related to functional language networks showed a negative connection with Broca’s area, though strongly left-lateralized, including in the left SFG/MFG (BA 6) and the precentral gyrus (BA 6 and 4), which may be involved due to the extended length of time spent in a relatively closed environment and the lack of spoken interaction. Cerri et al. (2015) suggested the mirror neuron system (MNS), including BA 6, is similar to monkey premotor area F5 (Gallese et al., 1996) and closely involved in articulatory rather than semantic speech. Hence, seafarers likely weakened motor control of speech production may be caused by a lack of opportunity to talk with each other randomly and frequently, under strict management with strong self-discipline consciousness. In contrast, the significant positive functional connections in the seafarers’ Wernicke’s area and the precuneus (including the posterior cingulate gyrus and the parietal) are the areas that are preferentially involved during the recall of real episodic memories rather than fictitious memories (Hassabis and Maguire, 2007). These findings probably indicate that seafarers have powerful cognitive functions which are able to recollect past professional experiences and predict future occurrences to make decisions for the future, including spatial navigation and use of the imagination that can contribute to seafarers’ career performance.

### 4.2. Language network’s lateralization and its relation to occupation

We examined the functional language networks’ lateralization of RSFC using Broca’s and Wernicke’s areas as independent seeds. According to the results of the language networks’ lateralization, functional language lateralization is related to some measures of *AI* asymmetry in seafarers and non-seafarers. We found slightly rightward lateralization in the Broca’s seed of seafarers with left IFG, and almost leftward asymmetric distribution in the non-seafarers (see Figure 4 and Table 2). Recently, studies have examined the lateralization of language networks with various elements (Nielsen et al., 2013; Chou et al., 2017; Schmitz et al., 2017). Schmitz et al. (2017) raised a novel perspective that genes related to language networks’ lateralization were specifically engaged in mental and neurological diseases. Moreover, while Bishop (2013) figured out that weak language lateralization may be the result of impaired language learning, other studies have suggested minimal involvement between the degree of language lateralization and performance (van Ettinger-Veenstra et al., 2010). The right-hemispheric activation might indicate additional resources are required for the process of integrating phonological input (van Ettinger-Veenstra et al., 2010). The precentral gyrus is related to exercise, and the cuneus and precuneus are involved in advanced cognitive functions. Here, seafarers have atypical language dominance, although a higher rate of atypical right hemispheric language lateralization was found in left-/mixed-handed people, and Packheiser et al. (2020) suggested that the assumptions related to language lateralization and dominant handedness need to be more deliberate. Furthermore, the atypia indicated that sentence processing was supported by the left and right networks (Labache et al., 2020), and semantic language performance was better (Bartha-Doering et al., 2018). Meanwhile, the lateralization of the left IFG was declared when processing iconic gestures with or without speech, overlapping with those brain regions that are also involved in advanced semantic information processing of speech (Özyürek, 2014). This may be a consequence of occupational skill-related requests: in the working environment of seafarers, it is necessary to communicate with foreigners and obey commands in various languages.

We also examined the occupational differences of functional language networks’ lateralization between two groups. The functional network of seafarers had ipsilateral and contralateral asymmetry located in the precuneus for both seeds. Furthermore, there was ipsilateral and contralateral asymmetry in the paracentral lobule and middle cingulum with Broca’s area as the seed, and also Wernicke’s ipsilateral asymmetry in the left SFG/MFG (see Figures 5, 6). Interestingly, a study on structural plasticity suggested that the left precuneus and paracentral lobule are closely related to spatial navigation training (Wenger et al., 2012), especially in young adults. Thus, the increased asymmetry of seafarers reflects the increased demand for professional competencies such as spatial navigation. Also, the right middle cingulate gyrus showed evidence that it is associated with general executive function in language conversion tests (Wang et al., 2007). Moreover, rsfMRI has shown the MFG is comparable with Broca’s area in its ability to determine hemispheric dominance for language (Gohel et al., 2019). Also, seafarers showed a significant increase in the left SFG and left MFG, which might relate to both gestures and spoken language used during voyages, as a few studies have found left MFG sensitivity to hand movements with unambiguous meanings (Willems et al., 2009). In summary, occupational factors have an impact on the functional language network of the brain.

### 4.3. Limitations and future works

This study is limited by sample size; studies in this area have yet to be conducted with larger datasets, and the robustness of the results should be treated with more caution due to the alternative steps in fMRI data processing (Murphy et al., 2009; Chai et al., 2012). In future, we are planning to recruit more subjects to explore and validate the findings regarding language networks and occupations. Due to the lack of behavioral data, such as detailed working years, we cannot clarify the relationship between language network and working years. More occupational research is needed, because occupation is a lifelong daily activity, and different occupations may have different effects on the brain language network; further research is needed in the future. Obviously, occupational effects on language networks occurs across the lifespan, and changes in the language network could be associated with various jobs. As a result, further work should be done on these points.

## 5. Conclusion

This study provides new findings that professional seafarers as a special occupation group elicited a weaker connection in the left SFG/MFG and left precentral gyrus with Broca seed-based RSFC, and a greater connection of Wernicke’s area with the cingulate and precuneus. Moreover, the slightly right-lateralized feature of functional language networks was observed in the Broca’s area of seafarers, but no significant voxels were observed in Wernicke’s area as seed; on the contrary, non-seafarers showed an almost leftward lateralization with Broca’s area as the seed, and rightward lateralization with Wernicke’s area. Interestingly, regarding the differences in language lateralization, the seafarers revealed greater connection with left Broca’s and left Wernicke’s areas. Overall, according to our findings, the seafarer’s occupation showed potential effects on brain language networks and their lateralization, which provides new evidence regarding occupational neuroplasticity and language.

## Data availability statement

The original contributions presented in this study are included in the article/supplementary material, further inquiries can be directed to the corresponding authors.

## Ethics statement

The studies involving human participants were reviewed and approved by the IRB of East China Normal University (ECNU). The patients/participants provided their written informed consent to participate in this study.

## Author contributions

HW and DP: conceptualization, methodology, validation, formal analysis, and writing—original draft. HY: conceptualization, methodology, validation, formal analysis, writing—original draft, and funding acquisition. YY: investigation and writing—review and editing. WZ: investigation, writing—review and editing, and data curation. CC and NW: conceptualization, resources, writing—review and editing, supervision, funding acquisition, and project administration. All authors contributed to the article and approved the submitted version.

## Footnotes

1
http://www.fil.ion.ucl.ac.uk/spm

2
http://www.restfmri.net

## References

[B1] Achard S. Salvador R. Whitcher B. Suckling J. Bullmore E. D. (2006). A resilient, low-frequency, small-world human brain functional network with highly connected association cortical hubs. *J. Neurosci.* 26 63–72. 10.1523/JNEUROSCI.3874-05.2006 16399673 PMC6674299

[B2] Agcaoglu O. Muetzel R. L. Rashid B. White T. Tiemeier H. Calhoun V. D. (2021). Lateralization of resting-state networks in children: Association with age, sex, handedness, intelligence quotient, and behavior. *Brain Connect.* 12 246–259. 10.1089/brain.2020.0863 34102875 PMC9058867

[B3] Bartha-Doering L. Kollndorfer K. Kasprian G. Novak A. Schuler A. L. Fischmeister F. (2018). Weaker semantic language lateralization associated with better semantic language performance in healthy right-handed children. *Brain Behav.* 8:e01072. 10.1002/brb3.1072 30298640 PMC6236252

[B4] Baxendale S. (2009). The wada test. *Curr. Opin. Neurol.* 22 185–189. 10.1097/WCO.0b013e328328f32e 19289955

[B5] Binder J. R. Frost J. A. Hammeke T. A. Cox R. W. Rao S. M. Prieto T. (1997). Human brain language areas identified by functional magnetic resonance imaging. *J. Neurosci.* 17 353–362. 10.1523/JNEUROSCI.17-01-00353.1997 8987760 PMC6793702

[B6] Bishop D. V. (2013). Cerebral asymmetry and language development: Cause, correlate, or consequence? *Science* 340:1230531. 10.1126/science.1230531 23766329 PMC4031634

[B7] Biswal B. Zerrin Yetkin F. Haughton V. M. Hyde J. S. (1995). Functional connectivity in the motor cortex of resting human brain using echo-planar MRI. *Magn. Reson. Med.* 34 537–541. 10.1002/mrm.1910340409 8524021

[B8] Bradshaw A. R. Bishop D. Woodhead Z. (2017). Methodological considerations in assessment of language lateralisation with fMRI: A systematic review. *PeerJ* 5:e3557. 10.7717/peerj.3557 28713656 PMC5508809

[B9] Broca P. (1861). Remarques sur le siège de la faculté du langage articulé, suivies d’une observation d’aphémie (perte de la parole). *Bull. Mem. Soc. Anat. Paris* 6 330–357.

[B10] Broday-Dvir R. Malach R. (2021). Resting-state fluctuations underlie free and creative verbal behaviors in the human brain. *Cereb. Cortex* 31 213–232. 10.1093/cercor/bhaa221 32935840

[B11] Catani M. Jones D. K. Ffytche D. H. (2005). Perisylvian language networks of the human brain. *Ann. Neurol.* 57 8–16. 10.1002/ana.20319 15597383

[B12] Cerri G. Cabinio M. Blasi V. Borroni P. Iadanza A. Fava E. (2015). The mirror neuron system and the strange case of Broca’s area. *Hum. Brain Mapp.* 36 1010–1027. 10.1002/hbm.22682 25366580 PMC6869026

[B13] Chai X. J. Castañón A. N. Öngür D. Whitfield-Gabrieli S. (2012). Anticorrelations in resting state networks without global signal regression. *Neuroimage* 59 1420–1428. 10.1016/j.neuroimage.2011.08.048 21889994 PMC3230748

[B14] Chen Y. C. Xia W. Chen H. Feng Y. Xu J. J. Gu J. P. (2017). Tinnitus distress is linked to enhanced resting-state functional connectivity from the limbic system to the auditory cortex. *Hum. Brain Mapp.* 38 2384–2397. 10.1002/hbm.23525 28112466 PMC6866871

[B15] Chou P. H. Lin W. H. Li W. R. Huang C. M. Sun C. W. (2017). Reduced language lateralization in first episode schizophrenia: A near infrared spectroscopy study. *Prog. Neuropsychopharmacol. Biol. Psychiatry* 78 96–104. 10.1016/j.pnpbp.2017.05.001 28499897

[B16] Damoiseaux J. S. Rombouts S. A. R. B. Barkhof F. Scheltens P. Stam C. J. Smith S. M. (2006). Consistent resting-state networks across healthy subjects. *Proc. Natl. Acad. Sci. U.S.A.* 103 13848–13853. 10.1073/pnas.0601417103 16945915 PMC1564249

[B17] Fisher R. A. (1921). 014: On the “Probable Error” of a coefficient of correlation deduced from a small sample. *Metron* 1, 3–32.

[B18] Fox M. D. Greicius M. (2010). Clinical applications of resting state functional connectivity. *Front. Syst. Neurosci.* 4:19. 10.3389/fnsys.2010.00019 20592951 PMC2893721

[B19] Friederici A. D. (2011). The brain basis of language processing: From structure to function. *Physiol. Rev.* 91 1357–1392. 10.1152/physrev.00006.2011 22013214

[B20] Gallese V. Fadiga L. Fogassi L. Rizzolatti G. (1996). Action recognition in the premotor cortex. *Brain* 119 593–609. 10.1093/brain/119.2.593 8800951

[B21] Gao Y. Linke A. Jao Keehn R. J. Punyamurthula S. Jahedi A. Gates K. (2019). The language network in autism: Atypical functional connectivity with default mode and visual regions. *Autism Res.* 12 1344–1355. 10.1002/aur.2171 31317655 PMC7147485

[B22] Gohel S. Laino M. E. Rajeev-Kumar G. Jenabi M. Peck K. Hatzoglou V. (2019). Resting-state functional connectivity of the middle frontal gyrus can predict language lateralization in patients with brain tumors. *Am. J. Neuroradiol.* 40 319–325. 10.3174/ajnr.A5932 30630835 PMC6375738

[B23] Gurunandan K. Arnaez-Telleria J. Carreiras M. Paz-Alonso P. M. (2020). Converging evidence for differential specialization and plasticity of language systems. *J. Neurosci.* 40 9715–9724. 10.1523/JNEUROSCI.0851-20.2020 33168623 PMC7726546

[B24] Hassabis D. Maguire E. A. (2007). Deconstructing episodic memory with construction. *Trends Cogn. Sci.* 11 299–306. 10.1016/j.tics.2007.05.001 17548229

[B25] Hein G. Engelmann J. B. Vollberg M. C. Tobler P. N. (2016). How learning shapes the empathic brain. *Proc. Natl. Acad. Sci. U.S.A.* 113 80–85. 10.1073/pnas.1514539112 26699464 PMC4711838

[B26] Jouravlev O. Kell A. Mineroff Z. Haskins A. Ayyash D. Kanwisher N. (2020). Reduced language lateralization in autism and the broader autism phenotype as assessed with robust individual-subjects analyses. *Autism Res.* 13 1746–1761. 10.1002/aur.2393 32935455

[B27] Kringelbach M. L. Berridge K. C. (2017). The affective core of emotion: Linking pleasure, subjective well-being, and optimal metastability in the brain. *Emot. Rev.* 9 191–199. 10.1177/1754073916684558 28943891 PMC5604465

[B28] Labache L. Mazoyer B. Joliot M. Crivello F. Hesling I. Tzourio-Mazoyer N. (2020). Typical and atypical language brain organization based on intrinsic connectivity and multitask functional asymmetries. *eLife* 9:e58722. 10.7554/eLife.58722 33064079 PMC7605859

[B29] Lane R. D. Ryan L. Nadel L. Greenberg L. (2015). Memory reconsolidation, emotional arousal, and the process of change in psychotherapy: New insights from brain science. *Behav. Brain Sci.* 38:e1. 10.1017/S0140525X15000011 24827452

[B30] Lottman K. K. Gawne T. J. Kraguljac N. V. Killen J. F. Reid M. A. Lahti A. C. (2019). Examining resting-state functional connectivity in first-episode schizophrenia with 7T fMRI and MEG. *Neuroimage Clin.* 24:101959. 10.1016/j.nicl.2019.101959 31377556 PMC6677917

[B31] Martin M. V. Cho V. Aversano G. (2016). Detection of subconscious face recognition using consumer-grade brain-computer interfaces. *ACM Trans. Appl. Percept.* 14:7. 10.1145/2955097

[B32] Mayeux R. Kandel E. R. (1985). “Natural language, disorders of language, and other localizable disorders of cognitive function,” in *Principles of neural science*, eds Kandel E. R. Schwartz J. (New York, NY: Elsevier), 688–703.

[B33] Murphy K. Birn R. M. Handwerker D. A. Jones T. B. Bandettini P. A. (2009). The impact of global signal regression on resting state correlations: Are anti-correlated networks introduced? *Neuroimage* 44 893–905. 10.1016/j.neuroimage.2008.09.036 18976716 PMC2750906

[B34] Nenert R. Allendorfer J. B. Martin A. M. Banks C. Vannest J. Holland S. K. (2017). Age-related language lateralization assessed by fMRI: The effects of sex and handedness. *Brain Res.* 1674 20–35. 10.1016/j.brainres.2017.08.021 28830770

[B35] Nielsen J. A. Zielinski B. A. Ferguson M. A. Lainhart J. E. Anderson J. S. (2013). An evaluation of the left-brain vs. right-brain hypothesis with resting state functional connectivity magnetic resonance imaging. *PLoS One* 8:e71275. 10.1371/journal.pone.0071275 23967180 PMC3743825

[B36] Olulade O. A. Seydell-Greenwald A. Chambers C. E. Turkeltaub P. E. Dromerick A. W. Berl M. M. (2020). The neural basis of language development: Changes in lateralization over age. *Proc. Natl. Acad. Sci. U.S.A.* 117 23477–23483. 10.1073/pnas.1905590117 32900940 PMC7519388

[B37] Özyürek A. (2014). Hearing and seeing meaning in speech and gesture: Insights from brain and behaviour. *Philos. Trans. R. Soc. Lond. B Biol. Sci.* 369:20130296. 10.1098/rstb.2013.0296 25092664 PMC4123675

[B38] Packheiser J. Schmitz J. Arning L. Beste C. Güntürkün O. Ocklenburg S. (2020). A large-scale estimate on the relationship between language and motor lateralization. *Sci. Rep.* 10:13027. 10.1038/s41598-020-70057-3 32747661 PMC7398911

[B39] Penfield W. (2015). *Mystery of the mind: A critical study of consciousness and the human brain.* Princeton, NJ: Princeton University Press. 10.1515/9781400868735

[B40] Phillips N. Shatil A. Go C. Robertson A. Widjaja E. (2021). Resting-state functional MRI for determining language lateralization in children with drug-resistant epilepsy. *Am. J. Neuroradiol.* 42 1299–1304. 10.3174/ajnr.A7110 33832955 PMC8324270

[B41] Połczyńska M. Beck L. Kuhn T. Benjamin C. Ly T. Japardi K. (2021). Tumor location and reduction in functional MRI estimates of language laterality. *J. Neurosurg.* 135 1674–1684. 10.3171/2020.9.JNS202036 33799298 PMC8909357

[B42] Rolinski R. You X. Gonzalez-Castillo J. Norato G. Reynolds R. Inati S. (2020). Language lateralization from task-based and resting state functional MRI in patients with epilepsy. *Hum. Brain Mapp.* 41 3133–3146. 10.1002/hbm.25003 32329951 PMC7336139

[B43] Schilbach L. Hoffstaedter F. Müller V. Cieslik E. C. Goya-Maldonado R. Trost S. (2016). Transdiagnostic commonalities and differences in resting state functional connectivity of the default mode network in schizophrenia and major depression. *Neuroimage Clin.* 10 326–335. 10.1016/j.nicl.2015.11.021 26904405 PMC4724692

[B44] Schmitz J. Lor S. Klose R. Güntürkün O. Ocklenburg S. (2017). The functional genetics of handedness and language lateralization: Insights from gene ontology, pathway and disease association analyses. *Front. Psychol.* 8:1144. 10.3389/fpsyg.2017.01144 28729848 PMC5498560

[B45] Sepeta L. N. Berl M. M. Wilke M. You X. Mehta M. Xu B. (2016). Age-dependent mesial temporal lobe lateralization in language fMRI. *Epilepsia* 57 122–130. 10.1111/epi.13258 26696589 PMC4749038

[B46] Shen W. Tu Y. Gollub R. L. Ortiz A. Napadow V. Yu S. (2019). Visual network alterations in brain functional connectivity in chronic low back pain: A resting state functional connectivity and machine learning study. *Neuroimage Clin.* 22:101775. 10.1016/j.nicl.2019.101775 30927604 PMC6444301

[B47] Shi Y. Zeng W. Wang N. (2017). SCGICAR: Spatial concatenation based group ICA with reference for fMRI data analysis. *Comput. Methods Programs Biomed.* 148 137–151. 10.1016/j.cmpb.2017.07.001 28774436

[B48] Shi Y. Zeng W. Wang N. (2021). The brain alteration of Seafarer revealed by activated functional connectivity mode in fMRI data analysis. *Front. Hum. Neurosci.* 15:656638. 10.3389/fnhum.2021.656638 33967722 PMC8100688

[B49] Stipdonk L. Boon R. Franken M. van Rosmalen J. Goedegebure A. Reiss I. (2021). Language lateralization in very preterm children: Associating dichotic listening to interhemispheric connectivity and language performance. *Pediatr. Res.* 91 1841–1848. 10.1038/s41390-021-01671-8 34408271

[B50] Sulpizio S. Del Maschio N. Del Mauro G. Fedeli D. Abutalebi J. (2020). Bilingualism as a gradient measure modulates functional connectivity of language and control networks. *Neuroimage* 205:116306. 10.1016/j.neuroimage.2019.116306 31654763

[B51] Szaflarski J. P. Holland S. K. Schmithorst V. J. Byars A. W. (2006). fMRI study of language lateralization in children and adults. *Hum. Brain Mapp.* 27 202–212. 10.1002/hbm.20177 16035047 PMC1464420

[B52] Tomasi D. Volkow N. D. (2012). Resting functional connectivity of language networks: Characterization and reproducibility. *Mol. Psychiatry* 17:841. 10.1038/mp.2011.177 22212597 PMC3323720

[B53] van Ettinger-Veenstra H. M. Ragnehed M. Hällgren M. Karlsson T. Landtblom A. M. Lundberg P. (2010). Right-hemispheric brain activation correlates to language performance. *Neuroimage* 49 3481–3488. 10.1016/j.neuroimage.2009.10.041 19853040

[B54] Villar-Rodríguez E. Palomar-García M. Á Hernández M. Adrián-Ventura J. Olcina-Sempere G. Parcet M. A. (2020). Left-handed musicians show a higher probability of atypical cerebral dominance for language. *Hum. Brain Mapp.* 41 2048–2058. 10.1002/hbm.24929 32034834 PMC7268010

[B55] Wang N. Zeng W. Shi Y. Yan H. (2017b). Brain functional plasticity driven by career experience: A resting-state fMRI study of the seafarer. *Front. Psychol.* 8:1786. 10.3389/fpsyg.2017.01786 29075223 PMC5641626

[B56] Wang N. Chang C. Zeng W. Shi Y. Yan H. (2017a). A novel feature-map based ICA model for identifying the individual, intra/inter-group brain networks across multiple fMRI datasets. *Front. Neurosci.* 11:510. 10.3389/fnins.2017.00510 28943838 PMC5596109

[B57] Wang N. Wu H. Xu M. Yang Y. Chang C. Zeng W. (2018). Occupational functional plasticity revealed by brain entropy: A resting-state fMRI study of seafarers. *Hum. Brain Mapp.* 39 2997–3004. 10.1002/hbm.24055 29676512 PMC6866348

[B58] Wang N. Zeng W. Chen D. (2016). A novel sparse dictionary learning separation (SDLS) model with adaptive dictionary mutual incoherence constraint for fMRI data analysis. *IEEE Trans. Biomed. Eng.* 63 2376–2389. 10.1109/TBME.2016.2533722 26929024

[B59] Wang N. Zeng W. Chen L. (2012). A fast-FENICA method on resting state fMRI data. *J. Neurosci. Methods* 209 1–12. 10.1016/j.jneumeth.2012.05.007 22659001

[B60] Wang N. Zeng W. Chen L. (2013). SACICA: A sparse approximation coefficient-based ICA model for functional magnetic resonance imaging data analysis. *J. Neurosci. Methods* 216 49–61. 10.1016/j.jneumeth.2013.03.014 23563324

[B61] Wang N. Zeng W. Chen D. Yin J. Chen L. (2015b). A novel brain networks enhancement model (BNEM) for BOLD fMRI data analysis with highly spatial reproducibility. *IEEE J. Biomed. Health Inform.* 20 1107–1119. 10.1109/JBHI.2015.2439685 26054077

[B62] Wang N. Zeng W. Shi Y. Ren T. Jing Y. Yin J. (2015a). WASICA: An effective wavelet-shrinkage based ICA model for brain fMRI data analysis. *J. Neurosci. Methods* 246 75–96. 10.1016/j.jneumeth.2015.03.011 25791013

[B63] Wang S. Van der Haegen L. Tao L. Cai Q. (2019). Brain functional organization associated with language lateralization. *Cereb. Cortex* 29 4312–4320. 10.1093/cercor/bhy313 30561523

[B64] Wang Y. Xue G. Chen C. Xue F. Dong Q. (2007). Neural bases of asymmetric language switching in second-language learners: An ER-fMRI study. *Neuroimage* 35 862–870. 10.1016/j.neuroimage.2006.09.054 17324590

[B65] Weiller C. Jüptner M. Fellows S. Rijntjes M. Leonhardt G. Kiebel S. (1996). Brain representation of active and passive movements. *Neuroimage* 4 105–110. 10.1006/nimg.1996.0034 9345502

[B66] Wenger E. Schaefer S. Noack H. Kühn S. Mårtensson J. Heinze H. J. (2012). Cortical thickness changes following spatial navigation training in adulthood and aging. *Neuroimage* 59 3389–3397. 10.1016/j.neuroimage.2011.11.015 22108645

[B67] Wernicke C. (1874). *Der aphasische symptomencomplex: Eine psychologische studie auf anatomischer basis.* Breslau: Max Cohn & Weigert.

[B68] Willems R. M. Özyürek A. Hagoort P. (2009). Differential roles for left inferior frontal and superior temporal cortex in multimodal integration of action and language. *Neuroimage* 47 1992–2004. 10.1016/j.neuroimage.2009.05.066 19497376

[B69] Wu H. Yan H. Yang Y. Xu M. Shi Y. Zeng W. (2020). Occupational neuroplasticity in the human brain: A critical review and meta-analysis of neuroimaging studies. *Front. Hum. Neurosci.* 14:215. 10.3389/fnhum.2020.00215 32760257 PMC7373999

[B70] Yan C. Zang Y. (2010). DPARSF: A MATLAB toolbox for” pipeline” data analysis of resting-state fMRI. *Front. Syst. Neurosci.* 4:13. 10.3389/fnsys.2010.00013 20577591 PMC2889691

[B71] Yan H. Wu H. Chen Y. Yang Y. Xu M. Wang N. (2022). Dynamical complexity fingerprints of occupation-dependent brain functional networks in professional seafarers. *Front. Neurosci.* 16:830808. 10.3389/fnins.2022.830808 35368265 PMC8973415

[B72] Yan H. Zuo X. N. Wang D. Wang J. Zhu C. Milham M. P. (2009). Hemispheric asymmetry in cognitive division of anterior cingulate cortex: A resting-state functional connectivity study. *Neuroimage* 47 1579–1589. 10.1016/j.neuroimage.2009.05.080 19501172

[B73] Yao S. Zeng W. Wang N. Chen L. (2013). Validating the performance of one-time decomposition for fMRI analysis using ICA with automatic target generation process. *Magn. Reson. Imaging* 31 970–975. 10.1016/j.mri.2013.03.014 23587929

[B74] Zhu L. Fan Y. Zou Q. Wang J. Gao J. H. Niu Z. (2014). Temporal reliability and lateralization of the resting-state language network. *PLoS One* 9:e85880. 10.1371/journal.pone.0085880 24475058 PMC3901661

[B75] Zuo X. N. Xu T. Jiang L. Yang Z. Cao X. Y. He Y. (2013). Toward reliable characterization of functional homogeneity in the human brain: Preprocessing, scan duration, imaging resolution and computational space. *Neuroimage* 65 374–386. 10.1016/j.neuroimage.2012.10.017 23085497 PMC3609711

